# Neuronal Activity in the Sciatic Nerve Is Accompanied by Immediate Cytoskeletal Changes

**DOI:** 10.3389/fnmol.2021.757264

**Published:** 2021-10-27

**Authors:** Bossmat Yehuda, Tal Gradus Pery, Efrat Ophir, Tamar Blumenfeld-Katzir, Anton Sheinin, Yael Alon, Noy Danino, Eran Perlson, Uri Nevo

**Affiliations:** ^1^Department of Biomedical Engineering, The Iby and Aladar Fleischman Faculty of Engineering, Tel Aviv University, Tel Aviv, Israel; ^2^Department of Physiology and Pharmacology, Sackler School of Medicine, Tel Aviv University, Tel Aviv, Israel; ^3^Sagol School of Neuroscience, Tel Aviv University, Tel Aviv, Israel

**Keywords:** biophysics, mechanics, neuronal plasticity, beading, Calpain

## Abstract

Mechanical events and alterations in neuronal morphology that accompany neuronal activity have been observed for decades. However, no clear neurophysiological role, nor an agreed molecular mechanism relating these events to the electrochemical process, has been found. Here we hypothesized that intense, yet physiological, electrical activity in neurons triggers cytoskeletal depolymerization. We excited the sciatic nerve of anesthetized mice with repetitive electric pulses (5, 10, and 100 Hz) for 1 and 2 min and immediately fixed the excised nerves. We then scanned the excised nerves with high-resolution transmission electron microscopy, and quantified cytoskeletal changes in the resulting micrographs. We demonstrate that excitation with a stimulation frequency that is within the physiological regime is accompanied by a significant reduction in the density of cytoskeletal proteins relative to the baseline values recorded in control nerves. After 10 Hz stimulation with durations of 1 and 2 min, neurofilaments density dropped to 55.8 and 51.1% of the baseline median values, respectively. In the same experiments, microtubules density dropped to 23.7 and 38.5% of the baseline median values, respectively. These changes were also accompanied by a reduction in the cytoskeleton-to-cytoplasm contrast that we attribute to the presence of depolymerized electron-dense molecules in the lumen. Thus, we demonstrate with an *in vivo* model a link between electrical activity and immediate cytoskeleton rearrangement at the nano-scale. We suggest that this cytoskeletal plasticity reduces cellular stiffness and allows cellular homeostasis, maintenance of neuronal morphology and that it facilitates in later stages growth of the neuronal projections.

## Introduction

Neuronal electrical activity is a cascade of biochemical events driven by the passive influx and efflux of ions through voltage-gated channels (Hodgkin and Huxley, 1952). Action potentials are accompanied also by mechanical events that occur in the immediate time scale. In the immediate temporal scale, nano-scale deformations are accompanying the action potential (Iwasa et al., 1980) and heat is released (Tasaki et al., 1989). These mechanical events are minute, but they cannot be explained solely by an electro-chemical view of action potentials. The exact molecular mechanism that leads to this mechanical wave is yet unclear. A physical model of propagation of mechanical solitons was proposed by Heimburg and Jackson (2005). They describe how an action potential may lead to a phase transition of the lipid membrane, that reflects non-linear changes in multiple mechanical and thermodynamic properties (Heimburg and Jackson, 2005), including those described by Iwasa et al. (1980) and Tasaki et al. (1989). This phase transition propagates similar to an action potential, without being dispersed (soliton). Mussel and Schneider (2019) demonstrated theoretically that a propagating acoustic wave in the interface of a lipid membrane and the electrolytic extracellular medium can yield a pulse of electric potential that behaves similar to an action potential. The molecular mechanisms leading to these events are yet to be resolved, and so is their physiological role.

Electrical activity was shown to trigger cytoskeletal changes occurring in the long time scales. Alvarez (1979) demonstrated that a 2 h stimulation of the preganglionic cervical nerve in cats resulted in an increase in the density of microtubules. Hu et al. (2008) imaged cultured hippocampal neurons to show that increasing neuronal activity by repeated perfusion with high KCl medium, enhanced the invasion of microtubules into spines and lengthened the duration of these invasions. Mitsuyama et al. (2008) complemented this result with a similar model of acute hippocampal slices and showed that strong stimulation leads to elongation of microtubules into spines, thus enlarging their protrusions. Merriam et al. (2013) further established the role of Calcium, F-Actin, and Drebrin in regulating the growth of microtubules in spines in similar experiments.

In a set of separate experiments, it was shown that intense electrical activity may lead to beading of axons. In non-living physical systems, as fluids enclosed by a cylindrical membrane, beading is typically linked to a change in the mechanical forces acting on the cylinder. A stable cylindrical system reflects an equilibrium between the stiffness of the cylinder, its surface tension, and the pressure and volume of the enclosed fluid. Once the pressure increases, for example by fluid inflow, minimization of free energy favors an increase in the volume. Given a fixed surface area, volume can be maximized by deforming the cylinder into spheres (thereby the term “beading”). This can be facilitated by reduction of the stiffness of the cylinder (Clerk Maxwell, 1874). Similarly, in neurons, different conditions and manipulations lead to a dramatic segmentation of the neuronal projections into swollen sphere-like segments (beads) separated by long shrunken “necks” (Ochs et al., 1994). Beading was observed in a variety of neuronal pathologies and conditions related to cellular stress like ischemia (Murphy et al., 2008; Risher et al., 2010), spreading depression (Takano et al., 2007), and seizures (Zeng et al., 2007).

To complete this short review of activity related morphological changes, we should mention that, in even longer timescales, neuronal plasticity facilitates morphological changes related to neuronal growth and the formation of networks (Shefi et al., 2005).

All the above mentioned events are necessarily linked to cytoskeletal changes. Currently there is no model that links the mechanical events that occur in different spatio-temporal scales. A model of the relation between cytoskeletal dynamics and the cellular events should include the transition from short and less intense physiological activity to pathological stimulation, the role of different cytoskeletal components linked to the above mentioned dynamics, and the way these affect different cellular structures, as axons, dendrites, and their growth cones.

We hypothesize that electrical activity affects the cytoskeleton immediately. We assume that activity triggers cytoskeletal de-polymerization such that this effect serves a physiological role in reducing the neuronal stiffness thus facilitating the morphological changes that occur later. We present an *in vivo* experiment that is composed of electron-microscopy imaging and analysis of cytoskeletal changes in axons after electrical stimulation.

## Methods

### Electrophysiology

**FIGURE 1:**
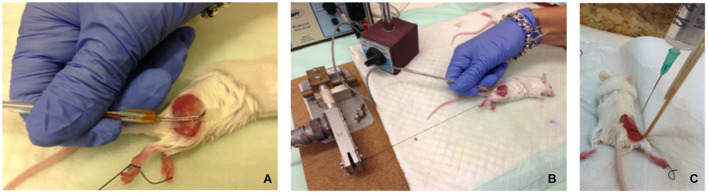
**(A,B)** Electrophysiological setup – exposed mouse sciatic nerve excited by electrical stimulus, using a hook electrode. **(C)** Fixation was performed *in situ* by constantly dripping Karnovsky fixative onto the exposed sciatic nerve for 30–40 min, while the mice were kept anesthetized.

**FIGURE 2:**
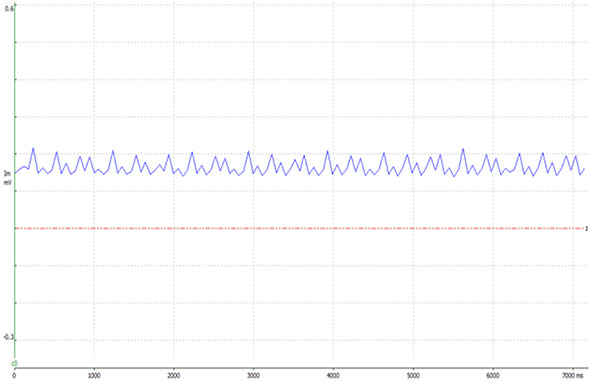
An example presenting evidence of the electrical stimulation, in the 10 Hz test group, using WinWCP. The graph shows data obtained from the force transducer, to which the leg of the mouse was tied. When stimulating the sciatic nerve, the leg contracts, elevating the voltage.

Electrical stimulation was applied to exposed sciatic nerves in male ICR mice (*n* = 17, 3 weeks old, weight: 35–38 gr) under anesthetic influence, using a hook electrode (Figures 1A,B). Nerves were excited at a frequency of 5 Hz for 1 min, 10 Hz for 1 or 2 min, and 100 Hz for 2 min. These stimulation frequencies (5 and 10 Hz) were chosen to be mimic intense physiological activity that is observed in mice sciatic nerve (Heglund et al., 1974; Herbin et al., 2018). Stimulation was verified by tying the leg with the exposed nerve to a force transducer and recording the mechanical contractions, which were also visible to the naked eye. An example of the force transducer recording is presented in Figure 2. As the stimulation frequency was higher, the mechanical contraction of the leg was more rapid. When stimulating at 100 Hz for 2 min, the leg was stiff, and the contraction was continuous. The same methods were used to expose and fixate the nerves of the mice in the control group, without any electrical stimulation.

### Fixation

Immediately after electrical stimulation, fixation was performed *in vivo* by constantly dripping 4% paraformaldehyde and 2.5% glutaraldehyde in a 0.1 M cacodylate buffer (pH 7.4) containing 3% sucrose (Karnovsky’s fixative) onto the exposed nerves for 15–20 min, while the mice were kept anesthetized (Figure 1C). The nerves were then excised, and all samples were fixed by immersion in the same fixative, on a shaker, for 60 min at 24°C. Next, each nerve was divided into segments of 2–3 mm (three to four segments per nerve) and fixed further by immersion in the same fixative, first at 24°C for 2–3 h and then at 4°C for over 24 h. This fixation protocol was chosen because it produced the best results, preventing cell death before completion of the fixation. The mice were kept alive during the fixation stage by using a special heated sheet to keep them dry and warm.

### Transmission Electron Microscopy

Tissues were rinsed four times, for 10 min each, in a cacodylate buffer and post-fixed and stained with 1% osmium tetroxide and 1.5% potassium ferricyanide in a 0.1 M cacodylate buffer for 1 h. Tissues were then washed four times in cacodylate buffer and dehydrated first in increasing concentrations of ethanol – 30, 50, 70, 80, 90, and 95% – for 10 min in each concentration; then three times in 100% anhydrous ethanol, for 20 min each time; and finally two times in propylene oxide, for 10 min each time. After dehydration, the tissues were infiltrated with increasing concentrations of Agar 100 resin in propylene oxide, consisting of 25, 50, 75, and 100% resin for 16 h for each concentration. The tissues were then embedded in fresh resin and placed in an oven at 60°C for 48 h for polymerization. Embedded tissues in blocks were sectioned with a diamond knife on a LKB III microtome, and ultrathin sections (80 nm) were collected on 200 mesh, thin bar copper grids. The sections were sequentially stained with uranyl acetate and lead citrate for 10 min each and viewed with a Tecnai 12 transmission electron microscopy (TEM) 100 kV (Phillips, Eindhoven, Netherlands), equipped with the MegaView II CCD camera and Analysis^®^ version 3.0 software (Soft Imaging System GmbH, Münster, Germany).

Random areas in slices of specimens from each group were examined and scanned. Images were acquired at magnifications of ×1,800 (for observation of large sections of the nerve), ×9,700 (for observation of a complete single projection transverse view), and ×37,000 (for cytoskeleton filament analysis). In the highest resolution (×37,000) multiple consecutive micrograph need to be imaged, in order to cover the entire axial section of each axon. Altogether 124 axons were blindly chosen for imaging with the highest resolution: control- 32; 5 Hz 1 min- 15; 10 Hz 1 min- 13; 10 Hz 2 min- 39; 100 Hz 2 min- 25.

### Image Processing

**FIGURE 3:**
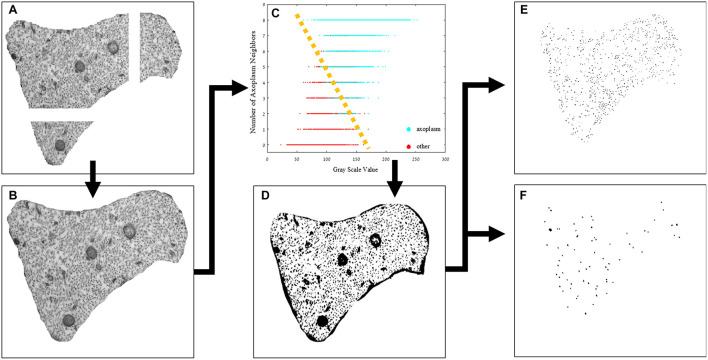
Image processing pipeline. **(A)** The original sub-images after normalization, **(B)** the original image combined using ImageJ^®^, **(C)** feature space of the designed classifier, **(D)** the axoplasm-and-cytoskeleton/organelles classified image, the two populations can be separated linearly, as illustrated in the image, and the labeled and separated images of intermediate filaments **(E)** and microtubules **(F)**.

Transmission electron microscopy images were analyzed to detect differences in the density of neurofilaments and microtubules across the different groups. Quantifying the cytoskeleton changes between the different groups required separation of the cytoskeleton components. Because the images produced by TEM are gray level with a range of average values of intensities in different regions of the cytoplasm, a simple threshold method did not achieve the best results. The method we used is detailed below and visually presented in Figure 3. Next, a set of parameters was chosen and extracted from the original images. Images were processed using MATLAB^®^ and ImageJ^®^. The image analysis is mostly automatic, and yet, to validate its results, we compared its outcome to the results obtained manually by independent users.

**FIGURE 4:**
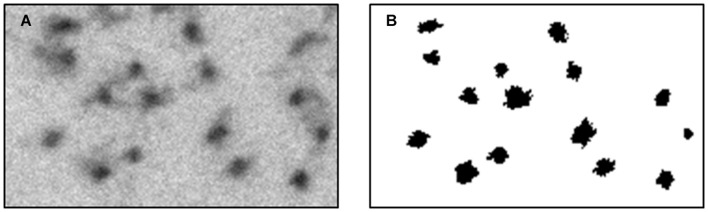
Classified sub-image, using the feature space designed. This process was conducted to confirm manually the automatic method. **(A)** a region from an original micrograph. **(B)** the classified binary image.

(1) All images (at a magnification of ×37,000) were normalized by a linear factor. This step is designed to prevent a shift of the original image-intensities histogram due to microscope artifacts or different lighting conditions present when each image was scanned. When observing an image histogram, a significant peak containing many of the image pixels can be distinguished in the image histogram. This bin represents the gray level of the axoplasm. To overcome the TEM scan differences, this gray level was set to a common, fixed level in all images and, therefore, each image had a normalization factor with a different value.
(2) The micrographs of each axon were combined using ImageJ software to create one complete axonal image, and its lumen was marked manually.
(3) A binary image was necessary for quantifying the changes in the cytoskeleton. We classified the image into two populations: the axoplasm and the cytoskeleton and organelles. Each pixel classification was determined using a specific feature space composed of (a) its’ normalized gray level and (b) the number of neighbors which were classified as lumen (from a 3 × 3 mask around each pixel). The data needed for assembling this space was collected from manually classified sub-micrographs of all experiment groups. Each pixel is represented as a feature vector. The feature space (in this case a plane) can be divided linearly into the two populations. With this feature space in place, each pixel in each new image is classified according to its properties, and a binary image is created according to this classification. Notice that a new image has no labeling therefore number of axoplasm neighbors cannot be calculated directly. to overcome this gap, a set of eight 3 × 3 masks was conducted (with all the possible divisions of axoplasm and cytoskeleton and organelles in it) and the classification was set according to the mask with minimal difference in gray level from the original image. An example of the classification method applied on a sub-image is presented in Figure 4.
(4) Extraction of the organelles and noise. Extraction of organelles was accomplished by identifying all the elements in the binary image that contained twice as many pixels as microtubules.
  Noise was removed by identifying all the elements in the binary image in which the number of pixels was half the number of pixels assessed for neurofilaments. In addition, other elements in the binary image were removed according to their roundness score. Roundness is defined as the normalized ratio between the circumference and the area of each object.

(5) The characteristics of microtubules and neurofilaments were quantified using the number of pixels that were classified for each of these cytoskeletal proteins. The number of pixels for each of the cytoskeleton components was determined according to the known average size (Jia et al., 2011) and the micrograph resolution.
(6) The following morphological features were quantified:

• Filament density normalized by cell size, separated into microtubules and neurofilaments.
• Nearest neighbor distance, which provides local measurements of the distribution of all the cytoskeleton components. This parameter is divided into the average value and a normalized histogram describing the distribution of values within the cells.
• Edge magnitude, which may indicate on the existence of cytoskeletal debris in the lumen, due to cytoskeleton depolymerization.

All data were expressed as the median value and the distribution of values between the first and third quartile with MATLAB^®^.

### Statistical Analysis

Normality of the distribution of each extracted parameter was tested using the Kolmogorov–Smirnov test. Most of the parameters where found to be distributed non-normally. To test the significance of differences across groups, we thus used the Kruskal–Wallis test, and whenever a statistical difference was found, we performed Mann–Whitney tests, with Bonferroni correction for multiple measurements, to find which are the groups that differ significantly.

To test the correlations between the different factors (given non-normal distribution), we used the Spearman correlation and then corrected the *P*-values for multiple comparisons, using the Benjamini–Hochberg (BH) correction.

## Results

**FIGURE 5:**
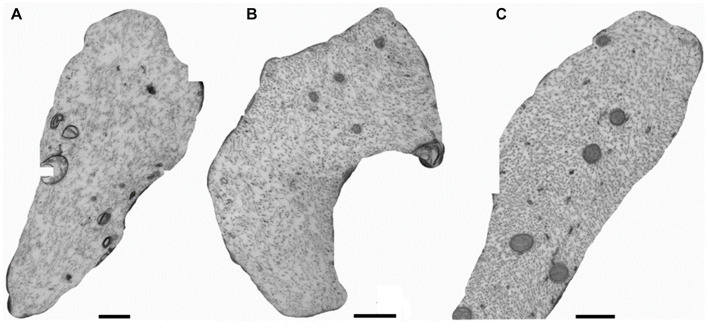
Cross sections of axons in the sciatic nerves of mice with electoral stimuli of 100 Hz **(A)**, of 10 Hz **(B)**, or in the absence of excitation (control group, **C**).

Representative micrographs of specific axons from the 100 Hz excitation (Figure 5A) and the 10 Hz (Figure 5B) excitation exhibit loss of densities of axial neurofilaments and microtubules from the lumen compared to the control non-excited group (Figure 5C).

**FIGURE 6:**
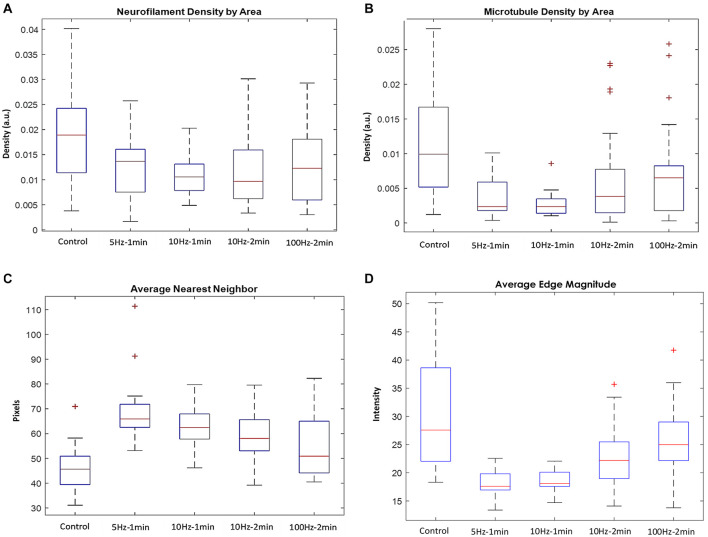
Quantified analysis of TEM micrographs. Density of **(A)** neurofilaments (*P* = 0.012 for the 10 Hz–2 min group, compared to the control) and **(B)** microtubules (*P* = 0.003, *P* = 0.001, *P* = 0.002 for the 10 Hz–2 min, 10 Hz–1 min, and 5 Hz–1 min groups, respectively), and **(C)** distances to the nearest neighbors (*P* = 0.00014, *P* = 0.00027, *P* = 7⋅10^– 7^, for the 10 Hz–2 min, 10 Hz–1 min, and 5 Hz–1 min groups, respectively). **(D)** The average edge magnitude in the micrographs.

A significant drop in the density of neurofilaments (Figure 6A) and microtubules (Figure 6B) was identified in the axon stimulated groups compared to the controls. For example, 10 Hz stimulations with durations of 1 and 2 min, resulted in neurofilaments density that dropped to 55.8 and 51.1% of the baseline median values, respectively. In the same experiments, microtubules density dropped to 23.7 and 38.5% of the baseline median values, respectively. Similar changes appear also in the 5 Hz stimulation group. However, results of the 100 Hz stimulation group had a much stronger variance, and the cytoskeletal densities dropped to more moderate values. The changes resulting due to stimulations with 10 and 5 Hz were statistically significant for both neurofilaments (*P* = 0.012 for the 10 Hz–2 min group, compared to the control) and for the microtubules (*P* = 0.003, *P* = 0.001, *P* = 0.002 for the 10 Hz–2 min, 10 Hz–1 min, and 5 Hz–1 min groups, respectively). Changes in the distances to the nearest neighbors, compared to controls were also statistically significant (*P* = 0.00014, *P* = 0.00027, *P* = 7⋅10^–7^, for the 10 Hz–2 min, 10 Hz–1 min, and 5 Hz–1 min groups, respectively).

Furthermore, the average distance of both microtubules and neurofilaments from their nearest neighbors significantly increased in the stimulated axons compared to the control (Figure 6C), indicating that these changes are local, throughout the entire axonal sections, rather than the outcomes of specific regions that bias the calculated averages. Interestingly, the control group and the group excited with a 100 Hz stimulation display the highest variability in the densities of the microtubules and neurofilaments and the distance to the nearest neighbor. No statistical significance was found for the comparison of the 100 Hz stimulation group with the control group.

**FIGURE 7:**
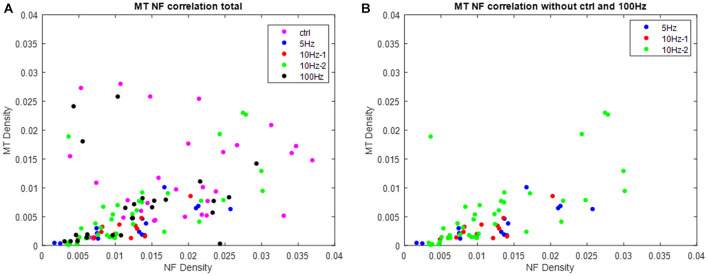
The correlation between the densities of microtubules and neurofilaments. Each point represents a single axon. **(A)** Densities of microtubules and neurofilaments from all axons (group color coded). **(B)** Densities of microtubules and neurofilaments from the axons stimulated with 5 and 10 Hz stimulations. Notice the significant correlation in these groups and the reduction in densities relative to the control group (*P* = 1⋅10^– 3^, *P* = 0.034, *P* = 1⋅10^– 7^, for the 5 Hz–1 min, 10 Hz–1 min, and 10 Hz–2 min groups, respectively).

Quantifying the average magnitude of the edges in the micrographs (in between cytoskeletal objects and the lumen) links the electric activity to the presence of electron-dense molecules, such as cytoskeletal debris in the lumen. Indeed, the sharpness of the edges is attenuated in the micrographs of the excited nerves (Figure 6D). The highest variability within axons of the same group was documented in the control group and the group of axons stimulated with 100 Hz. The substantial variability in these two groups is also reflected in the correlation between the densities of microtubules and neurofilaments within each axon, with more “outlayers” in the control and the 100 Hz groups (Figure 7) relative to the other stimulated groups. It should be noted that additional changes appear in the micrographs, especially in these of axons stimulated with a 100 Hz stimulation, on top of the quantified cytoskeletal changes. These include loss of mitochondria, loss of the integrity of the myelin sheath and intrusions appearing in the myelin sheath. Occurrence of changes in the benign stimulation frequencies (5 and 10 Hz) should be further studies.

## Discussion

We show with this *in vivo* model, that axon stimulation results in immediate rearrangements of the cytoskeleton, reducing the density of the cytoskeletal mesh. We suggest that the changes we identify in the density of neurofilaments and microtubules along the axons (Figure 6) result from activating a defined, highly regulated cytoskeleton cleavage process. We further suggest that this cleavage process is linked in a way that has never been related before to electrical activity. Action potentials are accompanied by an elevation in free calcium levels, both in the nerve cells and in their projections (Callewaert et al., 1996; Jia et al., 2011; Dana et al., 2019). It was further demonstrated in a model of neural axotomy that an elevation in free calcium levels induces activation of the Calpain protease that depolymerizes cytoskeletal proteins (Kamber et al., 2009). Moreover, depolymerization is pivotal in the creation of functional growth cones, following axotomy (Ziv and Spira, 1997). We suggest that a similar mechanism operates after electrical activity. Notice that Ca^+2^ concentration gradient across the cellular membrane exceeds these of other ions, since extracellular calcium concentration is ×10^4^ higher than that of the intracellular fluid (Jones and Keep, 1988). Once voltage gated channels open, the inflow of Ca^+2^ is very significant, and calcium transients can be detected even after a single action potential (Callewaert et al., 1996; Dana et al., 2019). Indeed, Hanemaaijer et al. (2020) demonstrated, with a model of pyramidal neurons, that single action potentials generate an immediate elevation in the calcium concentrations to near μM levels. The threshold for activation of μ-Calpain is Ca^2+^ concentration of 3–50 μM (Ma, 2013). Thus, these concentrations can be reached momentarily within a single train of action potentials and may trigger a proposed cascade of proteolytic activity – probably initiated in the cellular cortex – in a sub-second timescale. We suggest that in cases of repeated activity this depolymerization reduces the axonal stiffness. This initially results in axonal swelling and later on in beading. In even longer time-scales this activity-induced depolymerization facilitates a reduction of the stiffness that is a pre-requisite for growth. Our observation may thus reflect the first stage that can be followed later by axonal growth plasticity.

We observed changes in the density of microtubules that are opposite to some of the earlier results (Alvarez, 1979; Hu et al., 2008; Merriam et al., 2013). It is possible that the differences stem from differences in the experimental models used. However, it is also possible that we detected the initial outcome of the stimulated activity, whereas some of the earlier observations are related to the late dynamics of the cytoskeleton. Given this interpretation, we assume that the process observed here is a reversible physiological process. In low level stimulation it facilitates growth. In more intense stimulation that involves an increased intracellular pressure, it facilitates volumetric extension of the axon, thereby preventing rupture of its membrane. In either case, in vital cells it should be followed by re-polymerization.

Note that the mechanical response described here is linked to “trains” of multiple spikes and not to single action potentials. In addition, the reversible effect of repolymerization most likely occurs in much longer timescales of minutes to hours. Thus, the process described and suggested here may be related to the observations of a propagating mechanical wave (Iwasa et al., 1980; Heimburg and Jackson, 2005; Mussel and Schneider, 2019), but is different from these observations.

As noted, our results demonstrate that the most substantial effect occurs in the 5/10 Hz−1 min stimulation groups. We speculate that the relative elevation in density in the 100 Hz stimulations may be linked to the beading of axons that creates shrunken segments with densely packed cytoskeletal proteins (Ochs et al., 1994). The elevation in the variability of the 100 Hz stimulation group also supports this, as the imaged axonal sections may include swollen or shrunken segments.

Additional experiments that will involve long term tracking of the cytoskeletal dynamics should further test the proposed mechanism of depolymerization by Ca^+2^ and Calpain and the proposed morphological kinetics that occur in different time scales.

Finally, we should highlight also the work of Lin and Cantiello (1993) and of Priel et al. (2006) that observed that cytoskeletal elements (actin and microtubules, respectively) act as “transmission lines” or non-linear transistors that amplify the conducted electrical signal in the neuron. If our view of the cytoskeletal dynamics is correct there is a possible link between the de-polymerization and the adaptive nature of neuronal activity. This proposed link between Ca^+2^ triggered depolymerization and the results of Lin and Cantiello (1993) and of Priel et al. (2006) conforms with the observations that increase in Ca^+2^ level reduces firing frequency, and reduces the propagation velocity of action potentials (Lüscher et al., 1996).

To summarize, in this work we have demonstrated the immediate cytoskeletal changes that accompany neuronal excitation for the first time. Based on EM analysis, we suggest that electrical activity induced depolymerization of microtubules and neurofilaments. Awareness of this phenomenon may help expand our understanding of the biomechanical and chemical events during neuronal excitation. Our work may also have interesting neurophysiological implications on the understanding of the relationship between neuronal function and morphology as means of regulating adaptive neuronal activity, learning, and memory.

## Data Availability Statement

The original contributions presented in the study are included in the article/Supplementary Material, further inquiries can be directed to the corresponding author.

## Ethics Statement

The animal study was reviewed and approved by the Institutional Animal Care and Use Committee (IACUC) of Tel Aviv University.

## Author Contributions

UN initiated the study. UN, BY, EP, EO, and AS designed the experiments. BY, TG, EO, TB-K, and AS performed the experiments. BY, YA, ND, TB-K, UN, and EO performed the analysis. UN, BY, TB-K, and EP wrote the manuscript. All authors contributed to the article and approved the submitted version.

## Conflict of Interest

The authors declare that the research was conducted in the absence of any commercial or financial relationships that could be construed as a potential conflict of interest.

## Publisher’s Note

All claims expressed in this article are solely those of the authors and do not necessarily represent those of their affiliated organizations, or those of the publisher, the editors and the reviewers. Any product that may be evaluated in this article, or claim that may be made by its manufacturer, is not guaranteed or endorsed by the publisher.
