# Supplementary material for: Neuronal Activity in the Sciatic Nerve Is Accompanied by Immediate Cytoskeletal Changes
Source: Front Mol Neurosci. 2021 Oct 27;14:757264. doi: 10.3389/fnmol.2021.757264 (PMC8579013; doi:10.3389/fnmol.2021.757264)
Supplement: Supplementary file 1 [file Data_Sheet_1.pdf]

Supplementary Materials

Cytoskeletal changes following hypoxia

We observed the cytoskeletal changes in axons that originated from hypoxic conditions. These changes appear in axons of animals sacrificed before fixation and also in animals that die during fixation (after the process of fixation has already started). In both cases (see Figure S1) the cytoskeleton is completely broken and disordered. This phenomenon highlights two aspects of the process of cytoskeletal depolymerization that are of relevance to this and other works:

- **The continuum of damage inflicted to neurons**. Massive cytoskeletal damage leading to beading is a stage in the evolution of a neuron in response to insults. This beading increases the intracellular volume and prevents an immediate rupture of the swelling neuron. Clearly, if this condition persists, as in a prolonged intense electrical activity, this can be detrimental to the cell.
- **The importance of the dripping fixation procedure.** This procedure maintains the cytoskeleton and avoids creation of a severe experimental artifact.

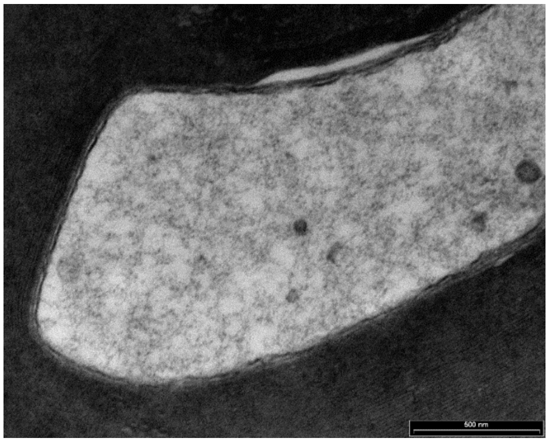

Fig. S1: An example of cytoskeletal breakage following hypoxia

Myelin intrusions

Figure S2 demonstrates the myelin intrusions that appear following intense electric activity. We attribute these intrusions to the process of shrinkage and beading.

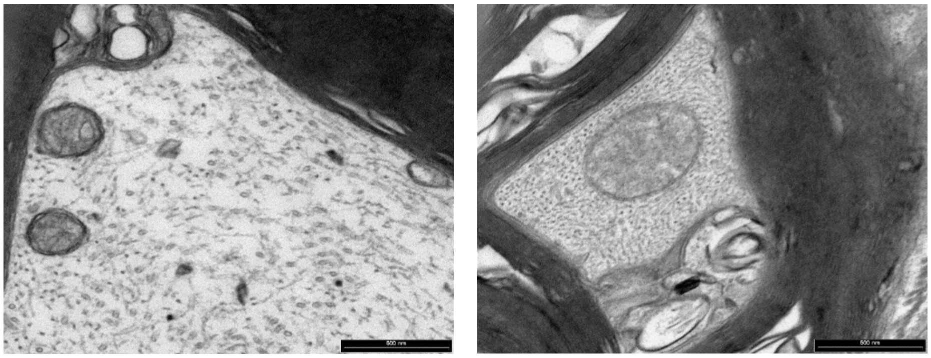

Fig. S2: Cross-sectional micrographs of axons with myelin intrusions. The left image was scanned after 100 Hz stimuli. The right image was scanned after 10 Hz stimuli
